# Metastatic Malignant Melanoma Mimicking Pancreatic Adenocarcinoma

**DOI:** 10.14309/crj.0000000000002119

**Published:** 2026-05-07

**Authors:** Sneh Sonaiya, Alexandra Jianu, Nicholas Jianu, Namrita George, Pei Xue, Rajan Amin, Vignan Manne

**Affiliations:** 1 Department of Internal Medicine, University of Nevada Las Vegas, Las Vegas, NV; 2 Department of Medicine, Lake Erie College of Osteopathic Medicine, Erie, PA; 3 Department of Gastroenterology & Hepatology, Kirk Kerkorian School of Medicine, University of Nevada Las Vegas, Las Vegas, NV

**Keywords:** metastatic melanoma, pancreatic metastasis, pancreatic mass, endoscopic ultrasound, gastroenterology

## Abstract

Malignant melanoma is an aggressive cutaneous malignancy with a high propensity for lymphatic and distant metastasis. While it commonly spreads to lymph nodes, lungs, brain, liver, and the gastrointestinal tract, pancreatic involvement is generally rare, occurring in less than 1% of cases. We report a case of metastatic melanoma initially misdiagnosed as pancreatic adenocarcinoma. This case underscores the importance of maintaining a broad differential, obtaining tissue diagnosis, and using immunohistochemistry to avoid diagnostic anchoring and ensure accurate identification of metastatic melanoma.

## INTRODUCTION

Malignant melanoma is an aggressive cutaneous malignancy with a strong propensity for regional lymphatic spread and distant metastasis. Its incidence continues to rise globally, affecting approximately 1 in 50 individuals in Western populations, with age-dependent and sex-dependent variations in incidence rates.^1,2^ Prognosis is highly stage-dependent, with 5-year survival exceeding 99% for localized disease but declining sharply to approximately 35% in the setting of distant metastases.^3^ Common sites of metastatic involvement include lymph nodes, lungs, brain, liver, and the gastrointestinal (GI) tract. Although autopsy studies demonstrate GI tract involvement in up to 50%–60% of patients with advanced melanoma, clinical diagnosis of GI metastases occurs in only 1.5%–4.4% of cases.^4,5^ Among GI metastases, pancreatic involvement is exceedingly uncommon, accounting for less than 1% of metastatic melanoma cases, with fewer than 200 cases of pancreatic metastases reported in the literature.^4,6–9^

Rarely, widespread melanoma may mimic other malignancies on imaging, such as pancreatic or GI cancers, and thereby, creating a diagnostic challenge. Early identification of pancreatic involvement as metastatic melanoma is crucial as it significantly influences prognosis and treatment. We present a rare case of metastatic malignant melanoma mimicking pancreatic adenocarcinoma to highlight this diagnostic pitfall and the importance of having a broad differential for patients who present with a pancreatic mass.

## CASE REPORT

**Figure 1.**
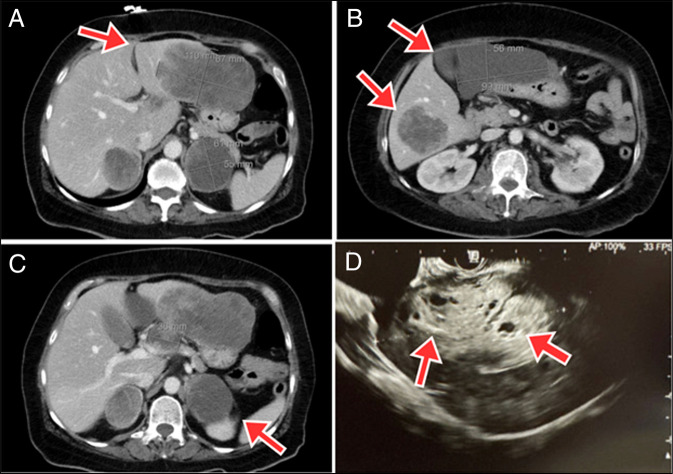
(A–C) Axial contrast-enhanced computed tomography images of the abdomen showing a large exophytic heterogeneous mass at the pancreatic head (3.7 × 3.2 cm) abutting the caudate lobe of the liver. Extensive metastatic disease is noted, including large lesions in the liver, bilateral adrenal glands, and retroperitoneum (red arrows). (D) EUS of the liver demonstrating multiple hypodense lesions (red arrows) consistent with hepatic metastases. Notably, no pancreatic mass or pancreatic ductal dilation was visualized on EUS. EUS, endoscopic ultrasound.

**Figure 2.**
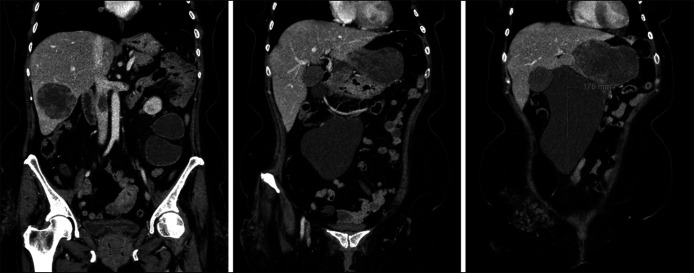
Contrast-enhanced computed tomography abdomen and pelvis (coronal views).

A 60-year-old woman with a history of hypertension and preexisting type 2 diabetes mellitus presented to the emergency department for 3 days of abdominal pain, distension, chills, and anorexia. Physical examination revealed lower abdominal tenderness and an irregular, violaceous growth on the right upper premolar gum line. On presentation, she was tachycardic to the 140s, prompting evaluation for thromboembolic complications. Computed tomography (CT) angiogram of the chest showed no acute pulmonary embolism but revealed innumerable solid pulmonary nodules diffusely throughout both lungs, concerning for a metastatic process. Contrast-enhanced CT of the abdomen and pelvis (performed without a dedicated pancreatic arterial-phase protocol) showed a 3.7 × 3.2-cm mass in the pancreatic head abutting the common hepatic artery and portal vein with an associated IVC thrombus and metastasis to the liver, lungs, subcutaneous tissue, lumbar spine, and adrenal glands (Figures 1 and 2). The tumor markers CA 19-9, CEA, and CA 27.29 were within normal limits. CA-125 was slightly elevated.

**Figure 3.**
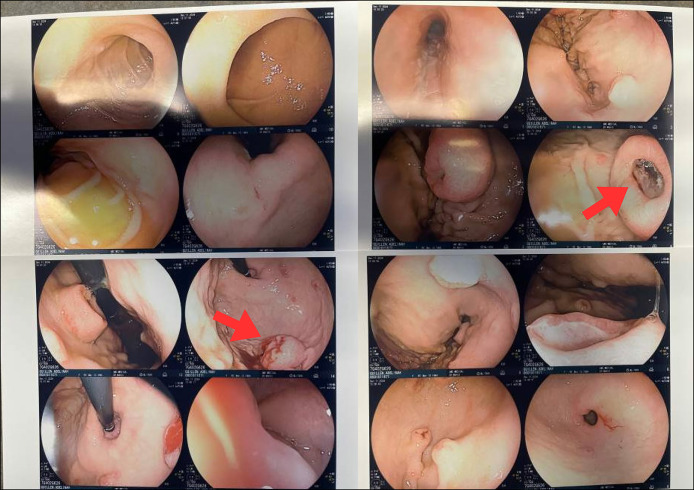
Esophagogastroduodenoscopy revealed multiple ulcerated gastric nodules (red arrows). These lesions were biopsied and, in conjunction with endoscopic ultrasound–guided liver biopsy, were histologically confirmed to be metastatic malignant melanoma.

Given the initial concern for metastatic pancreatic adenocarcinoma, the patient underwent esophagogastroduodenoscopy with endoscopic ultrasound (EUS) and biopsy. Esophagogastroduodenoscopy showed multiple nodules within the gastric body, ranging from 3 mm to 2 cm in diameter, many of which demonstrated nonbleeding ulcerations with central necrosis (Figure 3). Biopsies were obtained from 2 of the larger gastric nodules. Diffuse gastropathy was noted throughout the stomach. No Dieulafoy lesions or arteriovenous malformations were identified. The esophagus and duodenum appeared normal. Given the clinical context, these gastric lesions were highly suspicious for metastatic disease.

Endoscopic ultrasound demonstrated normal pancreatic parenchyma, normal ampulla, and no evidence of a pancreatic mass or pancreatic ductal dilation. Common bile duct and pancreatic duct were normal in caliber. A large heterogeneous mass was identified in the region of the celiac axis, concerning for metastatic disease. In addition, an approximately 8-cm heterogeneous lesion with both solid and cystic components was visualized in the left hepatic lobe, and fine-needle biopsy was performed. The right hepatic lobe could not be adequately evaluated with EUS.

Histopathologic examination of both the hepatic fine-needle biopsy and gastric biopsy specimens demonstrated metastatic malignant melanoma. The tumor identified in the liver was histologically identical to that seen in the gastric specimens. Immunohistochemical staining of the liver specimen was positive for S100, SOX10, and pan-melanoma antigen. Representative immunohistochemical staining patterns for metastatic melanoma have been previously described in the literature.^10^ Tumor cells demonstrated focal weak staining for cytokeratin AE1/AE3. Additional immunostains, including CK7, CK20, TTF-1, PAX8, GATA3, EMA, p40, chromogranin, synaptophysin, and NSE, were negative. p53 immunostaining demonstrated a wild-type pattern. These findings supported the diagnosis of metastatic malignant melanoma involving both the liver and stomach. Ultimately, the patient was discharged with plan for outpatient oncology follow-up. Unfortunately, her clinical course progressed rapidly, and she passed away within 1 month of hospital discharge.

## DISCUSSION

The case highlights a rare presentation of metastatic melanoma initially suspected to represent pancreatic adenocarcinoma with distant metastasis on cross-sectional imaging. While metastatic pancreatic adenocarcinoma was a reasonable initial consideration given the apparent pancreatic head lesion, the overall pattern of disease was atypical for a pancreatic primary. The presence of widespread metastatic involvement—including the liver, lungs, subcutaneous tissue, lumbar spine, and adrenal glands as noted on CT imaging—along with the absence of pancreatic ductal dilation or a discrete intrapancreatic mass on EUS, argued against pancreatic adenocarcinoma as the primary source of metastatic disease. These findings instead favored a disseminated metastatic process from an alternative primary malignancy. While cross-sectional imaging may suggest a pancreatic mass, it cannot reliably distinguish primary pancreatic neoplasms from metastatic or peripancreatic disease, particularly in the setting of extensive metastatic burden. Similar diagnostic mimicry has been reported in other GI malignancies, including a case of metastatic melanoma to the liver initially misdiagnosed as colorectal cancer with synchronous hepatic metastases, highlighting the limitations of imaging alone in distinguishing primary from secondary GI tumors.^11^ In our case, EUS findings were crucial in questioning the initial diagnosis and prompting tissue sampling for definitive diagnosis.

The discordance between CT and EUS findings in this case likely reflects a peripancreatic lesion mimicking a pancreatic head mass on cross-sectional imaging. Lesions adjacent to the pancreas, including peripancreatic metastatic lymph nodes or retroperitoneal deposits, may appear intrapancreatic on CT due to obscuration of normal tissue planes. Similar cases have been reported in which large peripancreatic metastatic lymph nodes were initially interpreted as pancreatic masses on CT imaging.^12^ By contrast, previous studies evaluating metastatic melanoma to the pancreas have demonstrated that true intrapancreatic lesions are typically well visualized on EUS, often appearing as hypoechoic or heterogeneous masses.^13^ Notably, EUS may also detect focal pancreatic lesions not appreciated on CT imaging. In our case, EUS demonstrated normal pancreatic parenchyma without a discrete mass, arguing against a true pancreatic lesion and supporting the interpretation that the CT finding represented peripancreatic metastatic disease rather than a primary pancreatic tumor. This highlights the complementary role of EUS in distinguishing primary pancreatic neoplasms from adjacent nodal or metastatic disease.

Metastatic melanoma mimicking other disease processes is infrequently described in the literature. Reported cases include metastatic melanoma of unknown primary initially presenting as neurofibromatosis type 2, metastatic melanoma to the liver misdiagnosed as colorectal cancer with synchronous hepatic metastases, and melanoma metastasis to the biliary tract mimicking hilar cholangiocarcinoma.^11,14,15^ Together with the present case, these reports highlight the variable clinical presentations of metastatic melanoma and underscore the potential for diagnostic misclassification when the disease presents at atypical sites or resembles more common malignancies.

The diagnostic challenge is further compounded in cases of melanoma of unknown primary, a recognized but uncommon entity that accounts for a minority of melanoma cases, and is frequently associated with advanced metastatic disease at presentation.^16^ In the absence of an identifiable primary lesion, metastatic melanoma may more readily mimic other malignancies, increasing the likelihood of delayed or incorrect diagnosis. This further underscores the importance of maintaining suspicion for melanoma even when no primary cutaneous lesion is initially apparent.

Although imaging modalities such as CT, magnetic resonance imaging, and positron emission tomography play a central role in staging melanoma—particularly in patients with stage III disease or high-risk stage II features—imaging findings are often nonspecific.^17–19^ As a result, histopathologic confirmation with immunohistochemistry remains essential for diagnosis. Melanoma characteristically expresses S100, SOX10, HMB-45, and pan-melanoma antigen, whereas epithelial markers such as cytokeratin AE1/AE3 support carcinoma, including pancreatic adenocarcinoma.^10,20,21^ In our case, tumor cells demonstrated strong positivity for S100, SOX10, and pan-melanoma antigen with focal/weak staining for AE1/AE3. This finding reflects a recognized phenomenon in which a minority of malignant melanomas exhibit aberrant cytokeratin expression.^22^ Importantly, this focal keratin staining does not alter the diagnostic interpretation when considered in the context of strong melanocytic marker positivity and the overall immunohistochemical profile, which remains consistent with malignant melanoma.

With the advent of immune checkpoint inhibitors and other immunotherapy-based treatments, early and accurate identification of metastatic melanoma has become increasingly critical to ensure timely initiation of appropriate therapy.^21^ In this case, immunohistochemical staining was pivotal in establishing the correct diagnosis and avoiding inappropriate surgical or cytotoxic treatment.

After the patient's passing, the family reported a previously unexamined cutaneous lesion that had not been recognized during the initial clinical evaluation. Although this finding could not be confirmed, it raises the possibility that the primary melanoma may have been cutaneous in origin. This case, therefore, underscores the importance of performing a thorough physical examination, including careful evaluation of the skin and mucosal surfaces, when metastatic melanoma is suspected. Maintaining a broad differential diagnosis when evaluating pancreatic masses—particularly in the setting of widespread metastatic disease and normal endoscopic evaluation of the pancreas—is essential to avoid diagnostic anchoring. Furthermore, the discordance between cross-sectional imaging and EUS findings in this case underscores the potential for peripancreatic metastatic disease to mimic primary pancreatic tumors on CT, while EUS may better delineate pancreatic parenchyma and clarify lesion origin. Accordingly, tissue diagnosis with immunohistochemistry remains essential for accurate classification. As therapeutic options for melanoma continue to evolve, timely and accurate identification of metastatic disease remains critical to guiding treatment decisions and optimizing patient outcomes.

## DISCLOSURES

Author contributions: S Sonaiya: conceptualization, writing original draft, resources, visualization, project administration. A. Jianu, N. Jianu, N. George: writing original draft. P. Xue: writing – review & editing, resources. R. Amin: writing – review & editing. V. Manne: writing – review & editing, resources, validation, supervision. S. Sonaiya and V. Manne are the article guarantors.

Financial disclosure: None to report.

Previous presentation: Previously presented at the American College of Gastroenterology Annual Scientific Meeting, October 25–29, 2025, Phoenix, Arizona.

Informed consent could not be obtained as the patient was deceased and the family could not be contacted despite reasonable efforts. All patient information has been fully de-identified, and this case report contains no identifiable protected health information.

## ABBREVIATIONS:

CT: computed tomography
EGD: esophagogastroduodenoscopy
EUS: endoscopic ultrasound
GI: gastrointestinal
IVC: inferior vena cava
MRI: magnetic resonance imaging
MUP: melanoma of unknown primary
PET: positron emission tomography
